# Circadian Disruption and Occupational Toxicants Exposure Affecting the Immunity of Shift Workers During SARS CoV-2 Pandemic

**DOI:** 10.3389/fpubh.2022.829013

**Published:** 2022-03-22

**Authors:** Siti Hanisah Mohd Fuad, Norsham Juliana, Nor Amira Syahira Mohd Azmi, Nur Islami Mohd Fahmi Teng, Sahar Azmani, Izuddin Fahmy Abu, Srijit Das

**Affiliations:** ^1^Faculty of Medicine and Health Sciences, Universiti Sains Islam Malaysia, Nilai, Malaysia; ^2^Faculty of Health Sciences, Universiti Teknologi Majlis Amanah Rakyat(MARA), Shah Alam, Malaysia; ^3^Institute of Medical Science Technology, Universiti Kuala Lumpur, Kajang, Malaysia; ^4^Department of Human & Clinical Anatomy, College of Medicine and Health Sciences, Muscat, Oman

**Keywords:** circadian rhythm, occupational toxicants, shift workers, COVID-19, immunity

## Abstract

In several regions of the world, the recent Coronavirus Disease-2019 (COVID-19) pandemic outbreak increased morbidity and mortality. The pandemic situation disrupted many workers' previously established lifestyles. The main aim of the present review was to describe the circadian disruption and occupational toxicant exposure affecting the immunity of shift workers during the SARS CoV-2 pandemic. We retrieved pertinent published literature from the Google Scholar, PubMed, and Scopus databases. In the present review, we discuss the circadian rhythm involving the hypothalamic-pituitary-adrenal (HPA) axis at the molecular level, its disruption, occupational toxicant exposure causing immunomodulatory effects, and the role of immunity during the SARS CoV-2 pandemic. The severity of the progression of the viral infection depends on multiple factors affecting immunity. Hence, shift workers may need to be aware of those factors such as circadian rhythm disruption as well as occupational toxicant exposure. The timing of shift workers' energy intake is also important concerning the shift of the workers. The information in the present review may be important for all workers who are at risk during the pandemic. In the absence of any published literature related to association of circadian rhythm disruption with occupational toxicant exposure, the present review may have greater importance.

## Introduction

A cluster case of pneumonia with an unknown origin was reported in Wuhan, Hubei Province, China, at the end of December 2019 (1). Through isolation of the causative agent utilizing human airway epithelial cells from affected individuals, a novel coronavirus known as 2019-nCov (SARS-CoV 2) was found (2). The World Health Organization (WHO) formally termed it as unique Coronavirus Disease-2019 (COVID-19) on 11 February 2020. It was later declared as a worldwide pandemic when mortality and morbidity increased worldwide (3).

According to the WHO database, the total number of cases worldwide was 162,184,263 as of 16 May 2021, with a total number of 3,364,446 deaths (4). South-East Asia (53%) had the highest number of new cases in the seven days preceding the report, followed by the Americas (25%) (4). Meanwhile, there were 470,110 cases in Malaysia, with 1,902 deaths (4). Selangor had the highest confirmed cases (153,663), followed by Sabah (59,349) and Johor (50,419) (5). Despite non-pharmacological prophylactic measures implemented by the government, such as social distancing, mandatory rules on wearing masks in public areas, quarantine, and movement control order/restriction (6), the numbers continue to rise.

The majority of COVID-19 clusters found in a South-East Asian country like Malaysia were linked to workplace clusters, accounting for 52.3% of the cases (7). As a result, it is critical to comprehend the circumstances that influenced these workers' immunological status during the current pandemic. Workers in the healthcare and manufacturing industries were the most exposed to the virus (8, 9). While some of them worked during regular office hours, most of them were shift workers who worked on one of three shifts i.e. morning shift, evening shift, or night shift.

Immunity refers to the body's defense against any external organisms or toxins. Immunity is often compromised in shift workers. The severity of any infection depends on the immune status of the individual. The immune status can be affected by multiple factors, such as circadian disruption and exposure to certain toxicants. Keeping the above facts in mind, we focused on the circadian rhythm involving the hypothalamic-pituitary-adrenal (HPA) axis and its disruption, workplace toxicants that influence workers, and the function of immunity during the COVID-19 pandemic.

## Materials and Methods

This was a narrative review. An extant literature search for relevant articles was conducted in May 2021 using three databases: Google Scholar, PubMed, and Scopus. Although it was a narrative review, the studies were identified based on information available in the title, abstract, and keywords. Relevant search terms included “circadian rhythm,” “shift work,” “occupational toxicants,” “COVID-19,” “shift work and circadian rhythm,” “immunity and COVID-19,” “circadian rhythm and immunity,” “occupational toxicants and immunity.”

We took into account all publications from 2011 until 2021. However, as COVID-19 began in 2019, all papers related to COVID-19 were limited from 2019 until 2021. One hundred seven studies out of 115 were included and were limited to publications in the English language.

The literature search was conducted by SHMF, NASMA, NIMFT, and SA. Articles were only accepted if all reviewers agreed that the article was relevant and of high quality. Any disagreement regarding the selection of studies was resolved by consensus. If necessary, 2nd, 6th, and 7th authors (NJ, IFA, and SD) were also consulted.

## Results and Discussion

The results and discussion section depicted the spread of COVID-19, circadian rhythm and disruption, the role of body immunity and occupational toxicant exposure.

### Spread of COVID-19 Infection

The SARS-CoV-2 virus belongs to the Coronaviriniae family and is classified as a beta coronavirus. It is a large, enveloped, spherical, single-stranded RNA virus genome measuring around 30 kb (10). It comprises four structural proteins, including nucleocapsid, envelops, membrane, and spike glycoprotein (11). During viral infection, neutralizing antibodies block viral particles from interacting with host cells. This virus exploits its spike glycoprotein to prevent neutralizing antibodies from working, allowing it to penetrate host cells (12). The spikes also prevent infected cells from undergoing apoptosis, ensuring their survival until the virus replication cycle is completed (13).

The transmission of COVID-19 occurs from person to person through respiratory droplets or contact with the infected patient's body fluids, such as saliva, tears, blood, urine, feces, and cerebrospinal fluid, with the mucosal lining of another individual (14). For example, respiratory droplets can be produced when an infected individual talks, coughs, or sneezes. These respiratory droplets can travel within 1 m up to 8 m (15–17). Fomite transmission is also possible as the virus remains alive and infectious on surfaces for up to 4–7 days, depending on the material of the surface (18).

According to multiple reviews, shift workers are at a higher risk of being infected by COVID-19 (19–21). Workers working in shift-work-based roles are reported to be 1.8 to 2 times more likely to be infected by COVID-19 compared to their counterparts (20). The severity predictors of COVID-19 depend on several factors, including age, pre-existing comorbidities, and immunity (22). Older age is associated with higher mortality and certain comorbidities such as cardiovascular disease, chronic kidney disease, chronic respiratory diseases such as asthma and chronic obstructive pulmonary disease, diabetes mellitus, hypertension, immunosuppression, and obesity. These are associated with unfavorable clinical courses, increased risk of intubation, as well as death (23–32). Several studies have concluded that shift workers are at a higher risk of developing these comorbidities, which directly increases the risk of them developing more severe COVID-19 complications (33–37).

### Physiology of Circadian Rhythm in the Body, and Its Effect on Immunity Dysfunction

The circadian rhythm, also considered a biological clock, is a system that regulates and facilitates the ability of an individual to respond and adapt to daily changes in the external environment. It includes almost all physiological and behavioral functions in an organism (38, 39). It lasts for about 24 h before another cycle begins, affecting multiple systems, such as the cardiovascular, gastrointestinal, neurological, and immune systems (40–43). The circadian rhythm is reset daily by the master clock in the body, which is located in the suprachiasmatic nucleus (SCN) in the hypothalamus (39). The circadian rhythm regulation happens at the cellular, tissue, and systemic levels. It is synchronized with the environment by synchronizers (external factors) such as light-dark and fast-feed cycles (44). In addition, the master clock/pacemaker in the SCN regulates the peripheral clock using mediators such as hormones and neuronal signals.

Gene expression and protein production, which peak and trough according to the time of day, are regulated at the molecular level. This is accomplished by using two transcription-translation oscillator loops that can up-regulate and down-regulate gene expression in the nucleus and counter-regulate one another, resulting in the 24-h cycle (45–47).

The mammalian clock system revolves around three major physiological components: the input pathway, the master clock itself, and the output pathway (48). The input pathway transmits stimuli, such as light, through the retino-hypothalamic (RHT) tract to the master clock in the SCN. This, in return, transduces into neuronal and hormonal signals used to regulate the peripheral clock (49). In addition, a few elements can affect the circadian cycle, such as the sleep/wake cycle, temperature, fast/feeding cycle, and light/dark cycle (50). Thus, changes to any of these factors can cause misalignment of the circadian rhythm, resulting in circadian disruption.

A significant component regulated by the circadian rhythm is the HPA axis. The HPA axis is an interactive neuro-endocrine unit comprising the hypothalamus, pituitary gland, and adrenal gland (51). This axis is essential for preserving homeostasis, adapting to the external environment, and regulating human emotions and cognitive functions (43). Cortisol and melatonin are some of the most prominent endocrine manifestations of the HPA axis, showing the rhythmic cycle of this biological clock (42, 49). The adrenal glands secreted the cortisol into the bloodstream and reached its peak level in the morning around 7 a.m. until 8 a.m., halved by midnight, and becomes lowest around 2 a.m.−4 a.m. (43). It is known to have many functions in the body, such as metabolism regulation and stress response mediator functions (52). Meanwhile, melatonin is produced by the pineal gland located near the center of the brain, which increases in the evening, with a peak at night and the lowest in the morning (53, 54). Both cortisol and melatonin are deeply related to the sleep/wake cycle as melatonin serves as a time cue for darkness and promotes sleepiness.

In contrast, cortisol has a significant role in initiating wakefulness (43, 55, 56). As melatonin levels decrease with age, older adults are more prone to developing sleep disorders such as insomnia and sleep-related breathing disorders (57). Both these hormones' dysregulation is also associated with chronic illnesses such as obesity and type-2 diabetes mellitus (58–60). Obesity refers to any abnormal accumulation of fat which poses a risk to health, and according to WHO, a body mass index of >30, may be considered obese. Melatonin levels are negatively correlated with insulin and leptin concentrations (54). Impaired cortisol and melatonin signals have disrupted glucose metabolism, resulting in systemic insulin resistance, higher fat mass accumulation, and gaining body weight (61, 62). While studies regarding direct interactions between cortisol/melatonin and immunity or virus' proteins are few, individuals with chronic metabolic disease exhibit weaker immune activity and decreased endogenous antioxidant levels (63). As such, shift workers with circadian disruption are more susceptible to a more severe course of an event during the COVID-19 pandemic (63).

**Figure 1:**
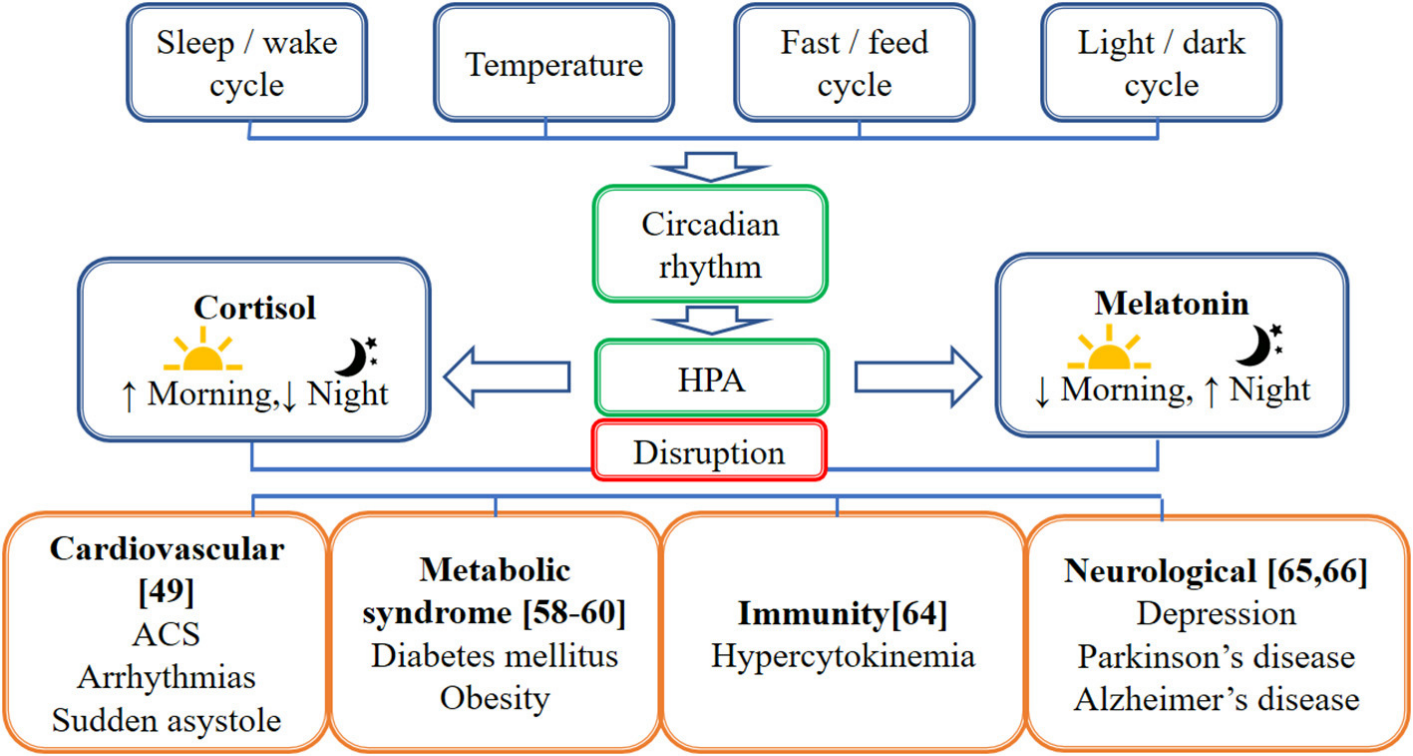
Schematic diagram on the factors affecting the circadian rhythm and the effect of circadian disruption on different body systems. This figure illustrates factors affecting the circadian rhythm which are the sleep/wake cycle, temperature, fast/feed cycle, and light/dark cycle. The hypothalamic-pituitary-adrenal axis (HPA) caused the cortisol to be high in the morning and lower in the night while melatonin is high in the night and lower in the morning. The circadian disruption affects different body systems, including cardiovascular, neurological, immunity, and metabolic functions.

**Figure 2:**
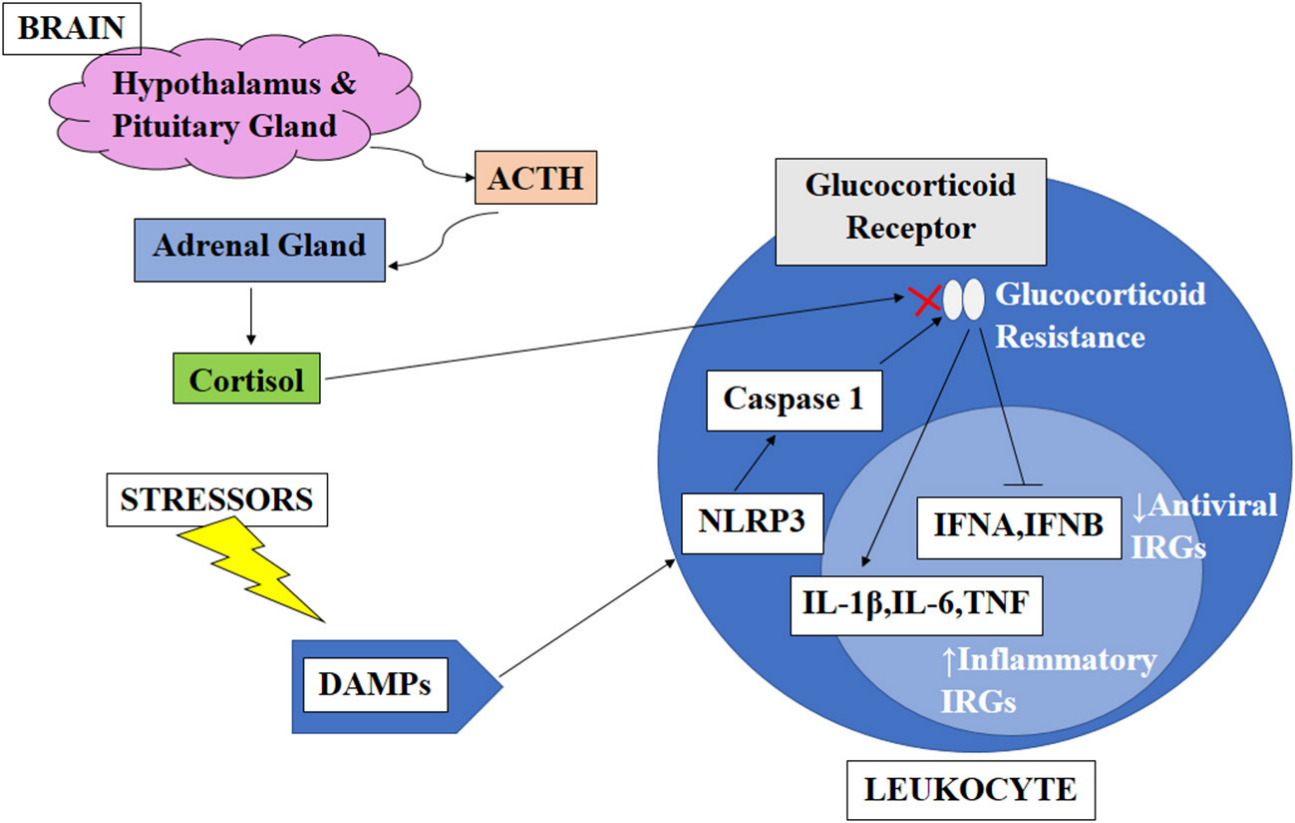
Schematic diagram on the cascading pattern of circadian disruption resulting in glucocorticoid resistance and immunity dysfunction. This figure illustrates the cascading pattern of circadian disruption resulting in glucocorticoid resistance and immunity dysfunction. The brain produces ACTH which caused the adrenal gland to release cortisol which has an anti-inflammatory effect by inducing a decrease in the production of interferon-γ (IFNγ), interleukin-2 (IL-2), and tumor necrosis factor (TNF). However, in circadian disruption, persistent activation of the HPA axis can induce glucocorticoid resistance of immune cells. Apart from that, psychological and physical stressors trigger the expression of endogenous damage-associated molecular patterns (DAMPs), which in turn activates the NOD-, LRR- and pyrin domain-containing protein 3 (NLRP3) inflammasome. These NLRP3 inflammasomes induce caspase-mediated cleavage of the glucocorticoid receptor, resulting in glucocorticoid resistance. Normally, both inflammatory and antiviral immune response genes (IRGs) are inhibited by cortisol. However, only antiviral IRGs are inhibited in the glucocorticoid resistance state, resulting in decreased expression of antiviral IRGs while increasing the expression of inflammatory IRGs (64).

An earlier study showed that cortisol has an anti-inflammatory effect by inducing a decrease in the production of interferon-γ (IFNγ), interleukin-2 (IL-2), and tumor necrosis factor (TNF) as measured by the percentage of CD4+ and CD8+ T-cells, which express these cytokines. However, in circadian disruption, persistent activation of the HPA axis can induce glucocorticoid resistance of immune cells. Although the mechanisms causing glucocorticoid resistance are unclear, one of the possible mechanisms proposed is the accumulation of the dominant-negative β-isoform of the glucocorticoid receptor driven by inflammatory cytokines. Apart from that, psychological and physical stressors trigger the expression of endogenous damage-associated molecular patterns (DAMPs), which in turn activates the NOD-, LRR- and pyrin domain-containing protein 3 (NLRP3) inflammasome. These NLRP3 inflammasomes induce caspase-mediated cleavage of the glucocorticoid receptor, resulting in glucocorticoid resistance. Normally, both inflammatory and antiviral immune response genes (IRGs) are inhibited by cortisol. However, only antiviral IRGs are inhibited in the glucocorticoid resistance state, resulting in decreased expression of antiviral IRGs while increasing the expression of inflammatory IRGs (64). Figure 1 illustrates factors affecting the circadian rhythm and the effect of circadian disruption on different body systems, including cardiovascular, neurological, immunity, and metabolic functions. Figure 2 illustrates the cascading pattern of circadian disruption resulting in glucocorticoid resistance and immunity dysfunction.

Shift workers are at a higher risk of developing diet-related chronic illnesses such as obesity, type 2 diabetes mellitus, and cardiovascular disease compared to non-shift workers (65–67). About 70% of shift workers are overweight/obese (67). This is due to the disruption of circadian rhythm, as discussed before, which is also attributed to their poor food habits. Shift workers are more prone to eating on a 24 h basis compared to non-shift workers, who usually eat three meals a day, with the food mostly being consumed during the daytime (49). The sleep constraint also increases the levels of leptin and ghrelin, thus increasing appetite at night. Apart from hunger, other factors also drive shift workers to eat at night, such as break and time availability, social pressure to eat with colleagues, avoiding gastric upset, stress eating, and a means to stay awake and prevent sleepiness (68). During overnight shifts, the circadian rhythm that regulates the body's internal processes is disrupted, which in turn causes dysregulation of the energy metabolism and promotes weight gain. The body is in a fasting state during night sleep, promoting the release of stored glucose as well as lower insulin sensitivity and insulin secretion compared to the daytime. As such, eating at night when the body is programmed to be in a sleeping state disrupts the metabolic processing standard (69).

### Occupational Toxicants Exposure in the Workplace and Its Effect on the Immune System

Occupational hygiene focuses on eliminating and controlling chemical, physical, or biological hazards. These hazards come in many forms, such as hazardous chemicals, gases, vapors, mist, dust, smoke, fumes, fibers, noise, vibration, heat, cold, ionizing radiation, non-ionizing radiation, and biological hazards such as bacteria, viruses, mold, or fungi. These workplace hazards are usually slow, and their effects are insidious on workers' health. There is an estimation of 2.4 million deaths linked to work-related illnesses annually throughout the world (70). Some of these toxicants have a detrimental effect on the immune system. In this pandemic, the industries with the highest exposure to COVID-19 are the factory and healthcare industries. The common types of occupational toxicants affecting the immune system are found in both industries, as summarized in Table 1.

**Table 1:** The types of occupational toxicants related to the immune system.

**References and country**	**Occupational toxicants**	**Occupational disease/condition**	**Mechanism of action**
Adeniyi et al. (71), Nigeria	Lead	Immunosuppression	Lead is an immunotoxicant that lowers humoral immunity, making the host more vulnerable to bacterial and viral infections.
Liangjiao et al. (72), China	Nanoparticles	Immunocompromisation	Nanoparticles interact with immunocompetent cells and induce immunotoxicity. In addition, it induces a pro-inflammatory response, oxidative stress and autophagy as the mechanism of toxicity.
Jagzape et al. (73), India	Bioaerosols	Asthma-like syndrome	Bioaerosols exposure can result in a variety of respiratory and mucosal symptoms ranging from mucous membrane irritation to acute or chronic disease. In addition, respiratory system reactions to bioaerosols may aggravate by different mechanisms, including nonspecific airway irritation, allergic reaction to antigens or inflammatory response.
Emara et al. (74), Saudi Arabia	Anesthetic gases	Immune dysfunction	Chronic inhalation of anesthetics results in a decrease in cellular antioxidant activity, decreased neutrophil function, and increased DNA breaks in lymphocytes.
Farahat et al. (75), Egypt	Ionizing radiation	Organ-specific autoimmune disorder	The impairment of cell-mediated immunity is linked to a rise in B-cell components and humoral immunity. There is a significant drop in CD4+ percentage levels with a shift of immune cells toward the cell-mediated immunity Th_1_.
Jia et al. (76), China	Formaldehyde	Allergic asthma, allergic contact dermatitis, immune diseases and cancer	Possible etiological mechanisms for airway inflammation induced by formaldehyde include oxidative damage, reactive oxygen species production, and increased histamine by eosinophilic activation. In addition, the predominance of humoral immunity as an immunological response to formaldehyde is confirmed by an imbalance of cytokine expression and a significantly higher ratio of B cells in peripheral blood.

### Role of Body Immunity During SARS-CoV-2 Infection

The human body has the ability, called immunity, to resist almost all types of organisms or toxins, as well as tend to damaged tissues and organs. Classically, the body's immune response is divided into innate and adaptive immunity. While innate immunity responds rapidly and non-specifically toward pathogens, adaptive immunity reacts slower but in a specific manner, producing a long-lived immunological memory (77). Furthermore, as the circadian rhythm regulates almost all aspects of physiological and behavioral response, the immune system is not excluded from this response and fluctuates daily (45). As such, the circadian rhythm regulates both innate immune response, including neutrophils, eosinophils, monocytes, natural killer (NK) cells, macrophages, mast cells, and dendritic cells, as well as adaptive immune response, which includes CD4+ and CD8+ T cells and B cells (78, 79).

Innate immunity is mediated by humoral systems and innate immune cells, such as myeloid cells, innate lymphoid cells, and NK cells. In contrast, adaptive immunity is based on the immunoglobulin family and cells such as T- and B- lymphocytes (80). Following a new pathogen infection, innate immunity is triggered first to eliminate the infection (81). They secrete pro-inflammatory cytokines that inhibit viral replication, recruit other immune cells to the site of infection, and stimulate the adaptive immune response (82). However, the initial clearance of the pathogen can fail due to the high virulence or number of invading pathogens. This is where adaptive immunity is activated to allow specific recognition and elimination of the pathogen, creating the immunological memory to respond more effectively during reinfection (83).

During the SARS-CoV-2 infection, the adaptive immune response can target the four proteins of the virus: envelope, spike glycoprotein, membrane, and nucleocapsid, as well as the addition of 20 non-structural replicase and regulatory proteins that the virus encodes to clear out the infection and develop protection (84). However, there can be a mismatched or delayed response between innate and adaptive immunity following COVID-19 infection (85). As such, an effective antiviral response of the host's innate and adaptive immunity is needed for the production of various pro-inflammatory cytokines and the activation of T cells, including CD4+ and CD8+, to stop viral replication and limit the spread of viruses, inflammation, and clean the infected cells (86). It was revealed that the hyperinflammatory response to SARS-CoV-2 infection was the major cause of the disease's severity and death (87). Compared to mild COVID-19 cases, the critical cases showed enriched chronic hyperinflammatory monocytes and depleted alveolar macrophages, which are known for their antigen-presenting characteristics (88). In mild COVID-19 cases, the adaptive immune response was more potent compared to the critical cases where there was a depletion of T-cells (88).

Individuals with diabetes or obesity were shown to have chronic inflammation and immune dysregulation by adipocytes, which produce an excessive number of cytokines. The increase in the number and hypertrophy of the adipocytes caused heavy stress on the adipose tissue, which in response released cytokines and chemokines such as interleukin-6 (IL-6) and monocyte chemoattractant protein (MCP-1) into the surrounding area. This, in turn, drew immune cells, especially macrophages, into the adipose tissue and subsequently promoted further expression of pro-inflammatory proteins such as IL-6, IL-8, IL-1β, and tumor necrotizing factor α (TNFα) (61, 89). This chronic inflammation and hypercytokinemia state subsequently lead to defective innate immunity (90). Adaptive immunity was also affected by obesity and diabetes, with a decline in naïve CD4+ T cells and imbalances between CD4+ T helper cells toward Th17 and Th22 pro-inflammatory subsets (91, 92).

**Figure 3:**
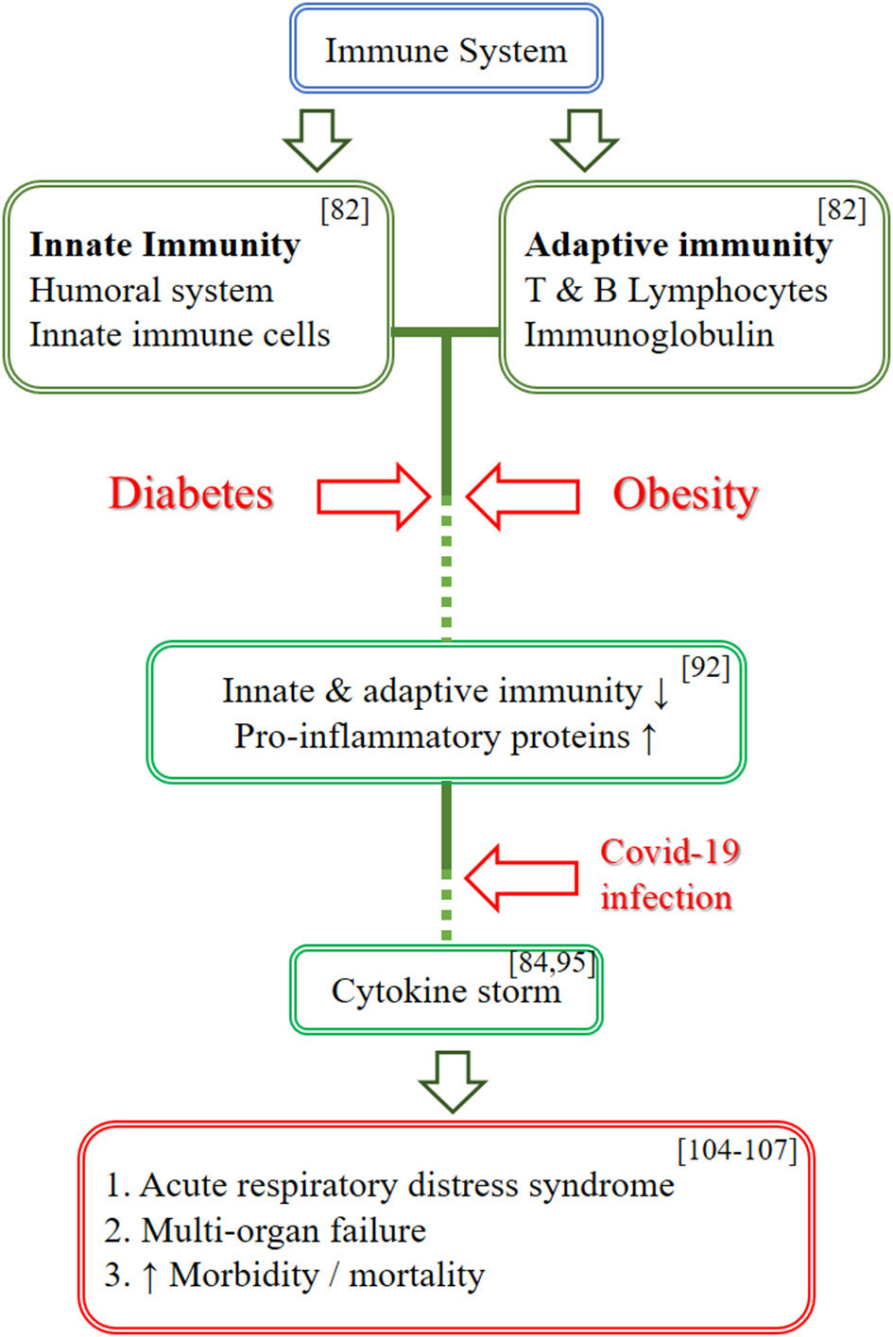
Schematic diagram on the effects of COVID-19 infection on the immune system in individuals with diabetes mellitus/obesity. This figure illustrates the effects of COVID-19 infection on the immune system in individuals with diabetes mellitus/obesity. The immune system is divided into innate and adaptive immunity. Diabetes and obesity caused the innate and adaptive immunity to be less affective and promote hypercytokinemia state as baseline by increasing the pro-inflammatory proteins. A COVID-19 infection further increases the cytokines level and leads to cytokine storm which in turn causing more severe complication such as ARDS and multi-organ failure, thus causing the increase in morbidity and mortality.

Individuals with circadian rhythm disruption have glucocorticoid resistance and metabolic diseases such as diabetes and obesity. As the baseline, these individuals with hypercytokinemia/hyperinflammatory state will cause COVID-19 to affect them more severely, leading to rather severe implications as it further increases the hypercytokinemia state, causing more severe implications in cytokine storm (82, 93). Figure 3 illustrates the effects of COVID-19 infection on the immune system in individuals with diabetes mellitus/obesity.

### Link Between Circadian Rhythm and Severity of COVID-19 and Its Clinical Significance

Interestingly, circadian rhythm plays an essential role in the severity of COVID-19 infection. The defense system in the body follows a rhythmic pattern. Hence, the relationship between circadian timekeeping, host immunity, and host-virus interactions is considered the latest concept (94). The host susceptibility to the microorganism was reported to be dependent on the biological clock in the body (95). Previous reports suggested that the time of the day of infection determines the severity of the progression of influenza, respiratory syncytial, and parainfluenza type 3 viruses (96–98). A detailed, in-depth study showed that the uptake of the SARS-CoV-2 virus in monocytes of the human body varies with the time of the day (95, 99). Following SARS-CoV-2 infection, there is an activation of the inflammatory response involving interleukin signaling and the complement cascade. Both processes are regulated by the circadian rhythm in the body (100).

Individuals with a disturbance in their circadian clock due to old age, working night shifts, or irregular sleeping and eating habits have a compromised immune system. As a result, they could be more susceptible to viral respiratory diseases (101). Future drug targets could also be planned according to the host factor of circadian oscillation.

## Conclusion

To summarize, immunity is an essential indicator of COVID-19 infection severity. An effective immunity response in innate and adaptive is important in any individual who are exposed to COVID-19 infection. The severity of infection is greater in individuals whose immunity is compromised. Immunity is affected by factors such as circadian rhythm misalignment and occupational toxicant exposure. As shift workers are more susceptible to circadian rhythm disruption and metabolic diseases, they are more likely to be infected by COVID-19 and develop rather severe complications such as ARDS, multi-organ failure, and death. There is a paucity of studies on the effect of occupational toxicant exposure on the immune system. Still, it is clear that occupational toxicant exposure worsens immunity in the long term. It is also not feasible to eliminate the shift work system in the current society in view of the expanding 24 h industry worldwide. In order to have the best possible work environment, the worker's health must be kept as a top priority in the employer's mind. Shift work is a modifiable risk factor for COVID-19 infection. Future studies must focus on suitable interventions for shift workers to reduce occupational toxicant exposure and circadian disruption. We also recommend future studies that should look into each worker's genetic polymorphisms and eventual epigenetic characteristics.

## Author Contributions

NJ and SD: conceptualization of the main idea of the manuscript and in charge of overall direction. SM: writing—original draft preparation and editing. NJ, SD, NASM, NIM, IA, and SA: supervision and provide critical feedback. All authors have read and agreed to the published version of the manuscript.

## Funding

This research was funded by Universiti Sains Islam Malaysia grants, grant numbers USIM/FRGS/FPSK/055002/50419 and USIM/MG/RSTECH/FPSK/055012/70619.

## Conflict of Interest

The authors declare that the research was conducted in the absence of any commercial or financial relationships that could be construed as a potential conflict of interest.

## Publisher's Note

All claims expressed in this article are solely those of the authors and do not necessarily represent those of their affiliated organizations, or those of the publisher, the editors and the reviewers. Any product that may be evaluated in this article, or claim that may be made by its manufacturer, is not guaranteed or endorsed by the publisher.
